# Pathogenicity of *Treponema denticola* Wild-Type and Mutant Strain Tested by an Active Mode of Periodontal Infection Using Microinjection

**DOI:** 10.1155/2012/549169

**Published:** 2012-07-05

**Authors:** Jacques Izard, Hajime Sasaki, Ralph Kent

**Affiliations:** ^1^Department of Molecular Genetics, The Forsyth Institute, Cambridge, MA 02142, USA; ^2^Department of Oral Medicine, Infection and Immunity, Harvard School of Dental Medicine, Boston, MA 02115, USA

## Abstract

The available passive mode of periodontal infections in mice requires high efficiency of bacterial attachment and invasiveness and is not always suitable to test the pathogenicity of genetically engineered mutant strains. We developed an active mode of oral infection, using microinjection in the marginal gingiva of mice, to test the pathogenicity of a genetically engineered *Treponema denticola* mutant strain deficient in intermediate-like filaments, compared to the wild-type strain. This targeted mode of infection inoculates the bacterial strain to be tested directly at a lesion site (needle entry point) located at the future periodontal lesion site. The efficiency of *T. denticola* wild-type strain to elicit bone loss contrasted with the lack of pathogenicity of the intermediate-like filament deficient mutant strain in comparison to the sham infection. The periodontal microinjection oral model in mice can be used for a variety of applications complementary to the passive mode of periodontal infection in context of pathogenicity testing.

## 1. Introduction

Progress in microbial oral diseases research has been impaired by the minimal amount of information available for most of the oral organisms and the limited availability of tools to study those organisms. In the context of over 600 common oral organisms associated with the human host from 13 phyla [1], only a few organisms have been investigated. With the unprecedented effort of the Human Microbiome Project both novel strains and genomic data are becoming available to the public [2–4]. While genetic tools are becoming available for a subset of organisms [5, 6], relevant animal models are still needed to study pathogenicity *in vivo* with the availability of engineered mutant strains [5, 7–10].

 Evaluating the pathogenesis of oral bacteria in animal model is a complex issue due to the multistep process involved. The bacteria arrive in the oral cavity associated with a solid (food for example) or a liquid (water for example) and have only a brief period in the oral cavity before entering the gastrointestinal tract. During that period, the bacteria have to “attach” to an oral surface that is comprised of covering cells (i.e., gingival, mucosal, or other host cells), a solid surface (tooth enamel or cementum), or other bacterial cells from the oral biofilms with which the bacteria can aggregate with [11]. This colonization step is also associated with population installation through cell division and competition with the present members of the oral flora, under the immunological pressure of the host.

 Two clinically relevant oral infections models are available in mice. The endodontic infection model provides a mono- or polyinfection model in absence of the oral biofilm competitors [12, 13]. This is an active mode of infection as a lesion is created to inoculated the bacteria [13]. The passive periodontal model of infection is based on the delivery of the bacteria within a liquid or a gel containing the bacteria [14, 15]. During the feeding, the bacteria adhere to an oral surface, compete with the oral microbiota, and are subject to host defenses.

 Studying engineered bacterial mutant strains in oral infection, including periodontitis, presents new challenges. While the mutation is targeted, the effect of such a mutation may be pleiotropic at the phenotype level when considering pathogenicity. Targeting specific component of a pathway may also alter the bacteria ability to colonize the gingiva. Competitiveness is a crucial element within a biofilm from colonization to population expansion. Are motility, attachment, and cell division rate critical factors when that particular bacterium is part of the biofilm?

 We propose an oral animal model using microinjection of bacteria in the gingival tissue. This mouse model allows to by-pass the initial flow, associated with ingestion, during which attachment each cell to tissue by providing a bacterial population in-situ at a site of lesion, which is the needle penetration site.

## 2. Material and Methods

This study was conducted in compliance with the approved animal protocol by the Institutional Animal Care and Use Committee at The Forsyth Institute (Animal Assurance Number A 3051-A1).

### 2.1. Bacterial Strains and Culture Method

*Treponema denticola* ATCC 33520 wild-type strain and the *cfpA*-interrupted mutant XC026 strain [7] were grown in New Oral Spirochete medium (NOS) with 10% heat-inactivated rabbit serum and 10 *μ*g of cocarboxylase per mL at 36°C in an anaerobic chamber (Coy Laboratory Products Inc., Grass Lake, MI) under an atmosphere of 85% nitrogen, 10% carbon dioxide, and 5% hydrogen [7]. Prior to inoculation, mid-log phase liquid cultures were spun down in the anaerobic chamber. Cell pellets were resuspended in anaerobic media. The cells were counted using a Petroff-Hausser slide following the manufacturer's protocol. Cells were maintained at 36°C under anaerobic condition, until injection.

### 2.2. Animals

 C57BL/6J male mice were purchased from the Jackson Laboratory (Bar Harbor, ME, USA). The mice were maintained under pathogen-free conditions. Five age-matched animals (age of 7 to 9 weeks) were used in this study per infection group. All animals received a powder diet immediately after weaning (from the age of 3 weeks) until being euthanized to avoid unreasonable alveolar bone loss induced by food impaction in between molars.

### 2.3. Intragingival Delivery of *T. denticola*

 All animals received antibiotic treatment (Sulfatrim suspension, 10 mL/100 mL of drinking water) for 4 days to reduce the original oral flora, followed by 3 days of an antibiotic-free period, prior to infection with *T. denticola*. On day 0, animals were anesthetized with 62.5 mg/kg ketamine HCl and 12.5 mg/kg xylazine in sterile PBS by intraperitoneal injection. After anesthesia, the mouse was mounted on a jaw retraction board to allow access to the oral cavity and the gingiva. A suspension of *T. denticola* (10^9^ cells/mL) was directly delivered into palatal interproximal papillae (1 *μ*L/site in 3 sites) of the maxilla. A surgical microscope enabled proper injection, with a 33G needle, in the marginal gingiva along the teeth without affecting the epithelial junction.

### 2.4. Measurement of Alveolar Bone Loss

 Animals were euthanized by CO_2_ inhalation on day 49 relative to infection. Maxillae were removed and hemisected. Hemisected maxillae were defleshed in a dermestid beetle colony [16], bleached, and mounted on a microscope slide for bone loss measurements. Palatal images of molar teeth and alveolar bone were captured using digital microscopy (Pixera Professional camera; Pixera, San Jose, CA). The measured polygonal area enclosed the cemento-enamel junction, the lateral margins of the exposed tooth roots, and the alveolar ridge. The measurements were performed using ImageJ [17], and calibration was performed using the image of a calibration standard at the same magnification (Edmund Optics, Barrington, NJ, USA). The alveolar bone loss was quantitated in a blinded manner. Results were expressed in mm^2^.

### 2.5. Statistical Method

Bone resorption (alveolar bone loss) measures on the left and right hemimandibles were averaged for each animal. Mean bone resorption was then computed for the five animals in each group. Mean levels in the three experimental groups were evaluated by one-way analysis of variance followed with pairwise comparisons by Tukey's multiple comparison procedure with overall type I error = 0.05. 

## 3. Results and Discussion

Microbial invasion and persistence in the oral cavity are a complex phenomenon. The newly implanted population is subject to a permanent immunological and mechanical challenge as well as a bacterial challenge form the environment and the food. In our experimental conditions, the bacterial challenge is limited to the coprophagic nature of the mice; water, food, and caging being sterilized.

 We have designed an active mode of marginal gingival infection allowing the testing of bacterial colonization at a site of injury. This infection site is in the gingival tissue affected by periodontal infection. The microinjection in the marginal gingiva is performed at higher dose than a passive infection, in only one application in contrast to other methods [14, 15]. This methodology is advantageous when studying genetically engineered mutant strains, where pathogenicity might be affected, and the step of *in vivo* infection alteration is unknown and might include attachment capabilities that are required to colonize the gingiva.

*T. denticola* wild type and the *cfpA* genetically engineered derived mutant strains were used to test the model of infection [7]. *T. denticola* is involved in the progression of periodontitis [18] and is known to be an invasive bacteria [19]. The *cfpA* mutant strain is deficient in intermediate-like cytoplasmic filaments, which are associated with cell division. The characteristic phenotypes are chromosome condensation and filamentation [7]. Another associated phenotype is a reduction in cell attachment [20]. Based on in vitro characterization, this mutant strain was hypothesized to be nonpathogenic. A passive model of infection would be inefficient to test the pathogenicity of the mutant strain, since it focuses on the ability of a strain to attach as a first step of colonization.

**Figure 1:**
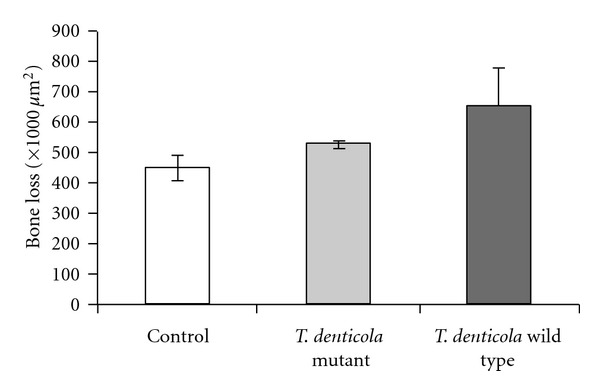
Bone resorption measured in wild-type mice infected by microinjection with *T. denticola* wild type, *cfpA* mutant strain, or the resuspending media (sham).

Gingival microinjection of *T. denticola* induced significant periodontal bone loss (Figure 1), the measured quantitative outcome of gingival infection and periodontal inflammation [15, 21]. In contrast, the *cfpA* mutant infected mice display a bone loss similar to the sham infection. The ANOVA procedure showed significant statistical difference between the three groups (*P* = 0.0056). The lack of infectivity of the *cfpA* mutant is underlined by the pairwise tests (Tukey procedure) indicating a significantly higher bone loss in wild-type mice related to the sham and *T. denticola cfpA* mutant groups. The drastic loss of infectivity might be originating from a lower fitness associated with the chromosome condensation as observed in vitro [7].

 Microinjection of wild-type and mutant strains or of bacteria that are known to sequentially coaggregate might circumvent the limitation of the passive modes of oral infection. The importance of the ultrastructure formed by the treponemal cytoplasmic filaments has been underscored both in vitro [7, 20, 22, 23] and presently *in vivo*. Their critical presence of intermediate-like filaments for *T. denticola* pathogenicity, their continuous length [22], and their single protein composition [24, 25] make them a candidate for drug-target development.
